# Be Careful

**Published:** 1855-10

**Authors:** M. Gowran


### BE CAREFUL.

BY M. GOWRAN.

Be careful, young man, when you first commence business, to choose such an occupation, as will be the most conducive to health; for it will be more valuable to you than silver or gold. Be careful, young lady, how you trifle with life or death, as though they were of no value; the course you are pursuing will endanger them in a great many ways. You often, in damp weather, promenade the street, with thin soled shoes, regardless of the consequences; besides, you attend balls and parties, in winter, wearing that kind of apparel which is only appropriate for summer. Then, if you exercise, so as to be a little too warm, you forget to *be careful*, and often sit or stand in a draught of cold air, until perspiration is checked—then the "bad cold," often leading to a "fatal disease." Add to this, late hours from novel reading, and late rising, too, which prevents the morning breeze from blowing gently around you, to give you new life and animate your depressed spirits. It's true, you may go on in this way for months, and perhaps for a few years: but remember, the day of reckoning will come; then you would retract, but it may be too late; for by this time the "hollow cough" begins to startle you; but you indulge the hope that it's only a "cold," and form the resolution to be more careful for the future. But what is past can never be recalled. Your disease may now baffle the skill of the most eminent physicians. Despite the ceaseless watchings and prayers of a kind-hearted father and an affectionate mother, the king of diseases performs his task; your form becomes almost a shadow, and you sink into a premature grave. But how often disease might be prevented! if we would *be more careful*, and attend to the laws of health. While penning these lines, we are carried by retrospection to the days of our youth, when, if we had been guided more by judgment, we might now be enjoying better health. But like many others, we are now trying to *be careful*, and to profit by the advice we receive from our physician. Let us hope that the subject of health, at no distant day, will be looked at in its true light, and receive the attention it deserves and demands, and not be so often sold for a few transitory pleasures.

College of Cookery.—It is proposed to open in London a "College of Domestic Economy," where everything necessary to the acquirement of a perfect knowledge of the culinary art and other domestic matters will here be taught by a person of great experience and acknowledged ability. The students for practice will be divided into classes of four or five each, with a servant-student to attend on each class, and assist them in their operations. Each class will provide what may be required for their practice, to be arranged by the student managing the class for the week; and the articles prepared will be consumed for their meals. No students will be admitted under fifteen years of age. Lectures will also be given to non-students, and likewise private instructions to ladies at their own residences.

If such colleges were instituted in this country, and conducted as they ought to be, and might be, and American girls who are poor and must work for a living, or do worse, infinitely worse, would enter them, and if a branch were added whose design was to prepare these young American girls for nursing children, a revolution would take place in domestic life more healthful and important than can well be conceived—a revolution, social, financial, moral, political and religious.

---

"Fish for Food.—Fish are said to be a very healthy food. With the exception of such as have oil interfused in their muscular tissues, fish are easy of digestion, and it is remarkable that fishermen and their families and those who consume a large quantity of fish, are healthy to a more than ordinary degree, and are almost wholly exempt from scrofula and pulmonary consumption."

This *fish story* is an evidence of the perfect carelessness with which newspaper articles as to health are thrown broadcast among the people, as millions of people have died without having been scrofulous or consumptive, in the interior of the country, away from the sea and large rivers, where fresh fish at the table is a rarity. It would be more rational to suppose that such remarkable exemption from the disease named was more probably the result of a plain mode of living combined with a life of out-door activity.—[Editor.

PICKLES.—"An excellent way to make pickles which will keep a year or more is—drop them into boiling hot water, but not boil them; let them stay ten minutes, wipe them dry, then drop them into cold spiced vinegar. They will not need to be put into salt and water."

---

TO KEEP CORN.—"The only way to keep sweet corn of any variety for winter use, is to partially cook and then dry it; or put it in a close jar, or other tight vessel. Corn nicely kept in this way is very good, as was abundantly tested, years before the Stowell corn was ever heard of."

---

TO KEEP APPLES.—"The most effectual method of preserving both apples and pears with which I am familiar—and which of course I recommend in preference to all others, is the following: Having selected the best fruit, wipe it perfectly clean and dry with a fine cloth, then take a jar of suitable size, the inside of which is thoroughly coated with cement, and having placed a layer of fine sand perfectly dry at the bottom, place thereon a layer of the fruit—apples or pears as the case may be—but not so close as to touch each other, and then a layer of sand; and in this way proceed till the vessel is full. Over the upper layer of fruit a thick stratum of sand may be spread and lightly pressed down with the hands. In this manner choice fruit perfectly ripe may be kept for almost any length of time, if the jar be placed in a situation free from moisture."

---

HOW TO MAKE STARCH FOR SHIRT-BOSOMS.—"Take two ounces of fine gum arabic powder, put it into a pitcher, and pour on a pint or more of boiling water, according to the strength you desire, and then, having covered it, let it stand all night; in the morning pour it carefully from the dregs into a clean bottle, cork it and keep it for use. A table-spoonful of gum-water stirred into a pint of starch made in the usual manner, will give to lawn, either white or printed, a look of newness, when nothing else can restore them after they have been washed."

